# Strengthening Mechanism and Carbide Precipitation Behavior of Nb-Mo Microalloy Medium Mn Steel

**DOI:** 10.3390/ma14237461

**Published:** 2021-12-05

**Authors:** Chunquan Liu, Fen Xiong, Yong Wang, Yuxin Cao, Xinbin Liu, Zhengliang Xue, Qichun Peng, Longsheng Peng

**Affiliations:** 1 Hunan Institute of Technology, Hengyang 421002, China; xiongfen93@163.com (F.X.); wangyong0911@wust.edu.cn (Y.W.); caoyuxin@wust.edu.cn (Y.C.); liuxinbin@hnit.edu.cn (X.L.); 2 The State Key Laboratory of Refractories and Metallurgy, Wuhan University of Science and Technology, Wuhan 430081, China; pengqichun1964@163.com; 3 Hunan Lifang Roll Co., Ltd., Hengyang 421600, China; penglongsheng@vip.163.com

**Keywords:** medium Mn steel, strength mechanism, nanometer-sized carbide, precipitation

## Abstract

This study investigates the strengthening mechanism and carbide precipitation behavior of medium Mn steel with Nb-Mo microalloy after cyclic quenching and austenite reverse transformation treatment. The results show that the Nb/Mo element not only precipitates (Nb,Mo)C in the grains, hindering the movement of dislocations and increases the strength, but also segregates at the austenite/ferrite grain boundary, thus delaying the transformation from austenite to ferrite. In addition, a large amount of nano-scale cementite is retained after cyclic quenching and austenite reverse transformation, which has a positive effect on the proportion of retained austenite in medium Mn steel. Moreover, the carbides with small size and low Mn content are dissolved, and the decomposed C and Mn content are beneficial to the nucleation of austenite during the intercritical annealing process at a temperature of 690 °C.

## 1. Introduction

With the intensification global energy crisis and environmental deterioration, countries around the world continue to attach importance to the research and development of lightweight and high-strength materials [1,2,3,4]. Medium-manganese steel has received widespread attention because of its high ultimate tensile strength and total elongation [5,6,7,8,9,10,11]. In recent years, more and more researchers have used Nb, V, Ti, Mo, and other elements for microalloying to further improve the strength of medium-manganese steel through main strengthening methods such as solid solution, fine grain, and precipitation. [12,13,14,15]. Nb can exist in the form of replacement atoms in medium Mn steel, and its atomic size of 15.56% larger than that of the iron. In addition, Nb tends to segregate at the grain boundaries and dislocations, and plays a strong drag role in the slip of dislocations, thereby hindering the recrystallization of austenite, increasing the recrystallization temperature of austenite and inhibiting the growth of austenite grains [16,17,18]. Simultaneously, as a strong carbon and nitrogen forming element, Nb forms carbon-nitrogen compounds with C and N during rolling process to pinning them at grain boundary, sub grain boundary, and dislocation, increasing the recrystallization temperature of austenite and prevent grain growth. In addition, the addition of Nb expands the temperature range of the non-recrystallized region, which can store sufficient deformation energy in the austenite before the phase transformation, and refine the ferrite grains during the phase transformation [19,20,21]. In order to highlight the role of Nb in medium Mn steel more effectively, the Nb-Mo compound addition method is usually used to strengthen the role of Nb in the steel. Additionally, the kinetics of carbide precipitation, and the transformation kinetics of ferrite as well as austenite can be changed by adding Mo [20]. Cao et al. [22] reported that during the coiling process of hot-rolled steel, Nb-Mo composite can better obtain the precipitation strengthening increment of microalloy carbides than Nb-Ti composite addition, and improve the yield strength of steel. Uemori et al. [23] reported that in refractory steels containing Nb and Mo, Mo can inhibit the growth and coarsening of NbC for a period of time after the segregation of Mo at the interface of NbC and the matrix, thereby increasing the high-temperature mechanical properties of the steel. Cai et al. [24] studied Fe-0.17C-6.5Mn-1.1Al-0.22Mo-0.05Nb medium manganese steel and found that the addition of Mo and Nb microalloys not only controlled the ratio of retained austenite and the mechanical stability during tensile deformation, but also has the effect of improving the yield strength.

The purpose of this study was to investigate the strengthening mechanism and carbide precipitation behavior in Nb-Mo microalloy medium Mn steel by using a cyclic quenching-austenite reverse transformation process. The strengthening mechanism of room temperature yield strength in medium manganese steel was clarified by analyzing the microstructure characteristics, precipitated phase morphology, and particle size distribution of medium manganese steel. In addition, the precipitation behavior of carbides in Nb-Mo microalloy manganese steel under cyclic quenching austenite reverse transformation treatment was discussed.

## 2. Experimental Procedure

**Figure 1:**
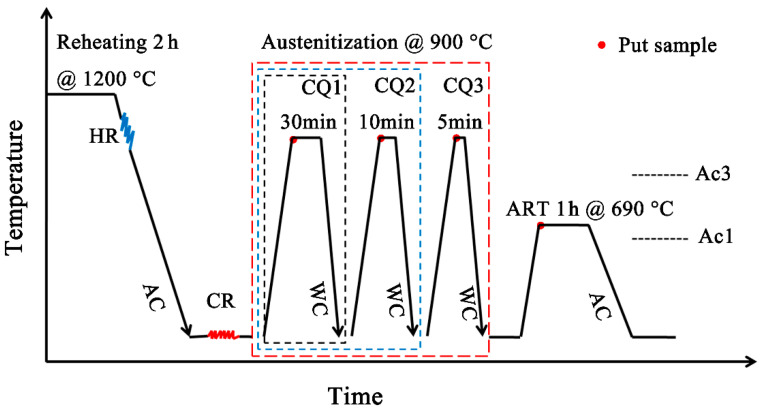
CQ-ART heat treatment process schematic diagram of cold-rolling experimental steel.

The investigated alloy has a nominal chemical composition of 0.25C-3.98Mn-1.22Al-0.20Si-0.19Mo-0.03Nb-Fe (wt.%). The steel was melted in 15 Kg intermediate frequency induction furnace and casted into an ingot, and then the ingot was forged to 100-mm wide and 30-mm (thick) slab. Afterwards, the slab was hot-rolled to 3.8-mm-thick plates after undergoing the solution treatment at 1200 °C for 2 h, and then cold rolled to 1.9-mm thick sheets. The AC_1_ and AC_3_ temperatures of the experimental steel were measured in our previous work [7]. The schematic diagram of the CQ-ART heat treatment process of the experimental steel is shown in Figure 1. Three processes of cold rolling samples were separately carried out as follows: For CQ1- sample, it was rapidly reheated to austenitization temperature at 900 °C for 30 min, and then water quenched to room temperature; for CQ2 sample, a similar cyclic quenching heat treatment was used for 10 min based on CQ1, and then water quenched to room temperature; with regard to the CQ2 process, the sample was rapidly reheated to 900 °C for 5 min, followed by being water quenched to room temperature; finally, three experimental steels treated with different processes with intercritical annealing at 690 °C for 1 h, respectively, and then air-cooled to ambient temperature.

The experiment samples of 12.5 mm width were cut using a wire electrode from heat-treated processed sheets along the rolling direction. Tensile tests were performed on a UTM5504GD tensile testing machine with the strain rate of 3 mm/min. Before SEM (FEI, Hillsboro, OR, USA) and TEM (JEOL, Tokyo, Japan) analyses the samples were etched in a 4 vol.% Nital and 96% C_2_H_5_OH mixed acid solution. The partition behavior of Mn was determined by TEM-energy dispersive spectrometry (TEM-EDS) (JEOL, Tokyo, Japan). Austenite content was determined by X-ray diffraction (XRD; Bruker, Karlsruhe, Germany) based on the integrated intensities of (200)α, (211)α, (200)α, (220)γ, and (311)γ diffraction peaks [6,7].

## 3. Results and Discussion

### 3.1. Microstructure and Mechanical Properties

**Figure 2:**
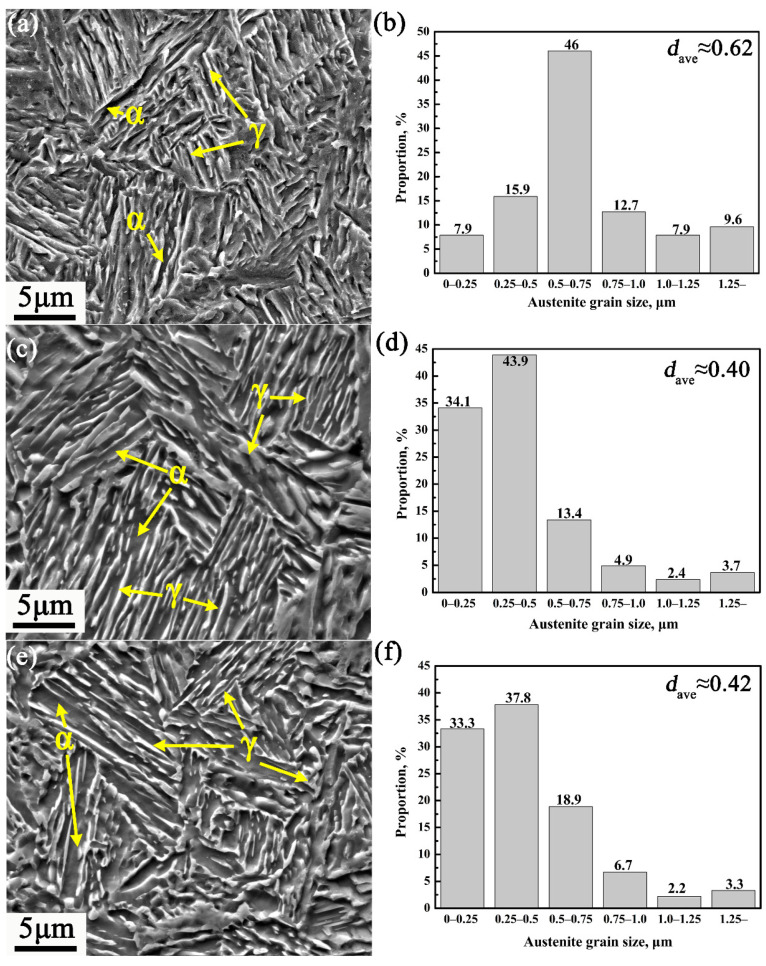
SEM, and grain size graphs of (**a**,**c**,**e**) and (**b**,**d**,**f**) for CQ-ART samples, respectively. (**a**,**b**) CQ1-ART sample, (**c**,**d**) CQ2-ART sample, and (**e**,**f**) CQ3-ART sample.

Figure 2a,c,e shows the typical SEM microstructure of cyclic quenching austenite reverse transformation. Obviously, the microstructure of the three samples mainly consisted of strip-shaped retained austenite and ferrite, while the microstructure of the CQ2-ART sample is mainly composed of relatively short strip-shaped retained austenite and ferrite (see Figure 2c). At the same time, it was observed that nano-size carbides are found in all three samples after cyclic quenching, which are rod-shaped and spherical. With increasing of quenching times, the shape of the carbide gradually evolved from a rod shape to a spherical shape. Previous studies have shown that a large number of cementite particles will be formed in the medium-manganese steel under the intercritical annealing treatment with a short time [25,26]. These cementite particles may be formed during the heating process, then in the subsequent intercritical annealing process, for one thing, the cementite particles served as nucleation sites for new austenite grains; for another, these cementite particles usually contain high C and Mn contents, which may result in enhanced chemical stability of nucleated austenite grains during intercritical annealing dissolution, or reduced stability when insoluble; the former may result in the formation of more austenite grains with finer size during the intercritical annealing, while the latter may lead to less retained austenite because of the reduced austenite grains chemical stability [27]. Austenite grain size was characterized by Digital Micrograph software and using 30 TEM images. The measured width of the retained austenite in the CQ1-ART, CQ2-ART, and CQ3-ART samples was 0.16~1.85 μm, 0.13~1.25 μm, and 0.12~1.60 μm, respectively (see Figure 2b,d,f). Obviously, as the number of cyclic quenching increased, the average width of strip-shaped retained austenite decreased from 0.62 to 0.40 μm. Analysis indicates that the cyclic quenching process hinders the austenite recrystallization and growth process, then finer original lath martensite is obtained, and finally fine strip retained austenite nucleating along the martensite in the subsequent reverse austenite transformation process is obtained [7]. It is worth noting that rod like particles of 30–100 nm and a part of coarse cementite particles are found in the three samples, which contain from 28 wt% to 7 wt% Mn in the austenitic phase.

**Figure 3:**
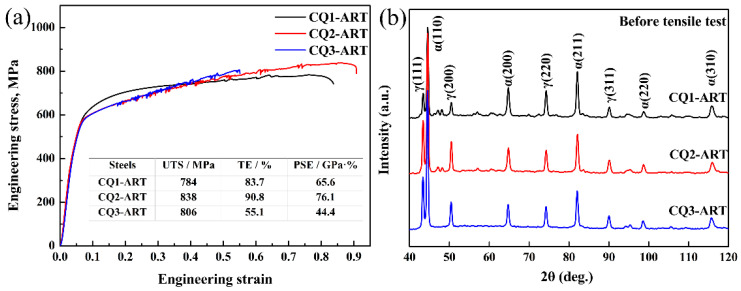
(**a**) Engineering stress–strain curves and (**b**) XRD patterns.

Engineering stress–strain curves of three cyclic quenching austenite reverse transformation samples are shown in Figure 3a. It can be seen that there is no obvious yield platform in the curve. For CQ1-ART sample, its ultimate tensile strength (UTS) of 784 MPa, the total elongation (TE) of 83.7%, and the product of strength and elongation (PSE) of 65.6 GPa%; for CQ2-ART sample, it is characterized as: the UTS of 838 MPa, the TE of 90.8%, and the PSE of 76.1 GPa%; for CQ3-ART sample, the UTS of 806 MPa, while TE decreased by 55.1% compared to CQ1-ART. The tensile strength of the three samples changed little, while total elongation increased initialy and then decreased sharply with the increase of cyclic quenching times. Figure 3b shows the XRD patterns of three CQ-ART samples. As shown in Figure 3b, with the increasing of the number of cyclic quenching, the austenite diffraction intensity peaks of γ(200), γ(220), and γ(311) have a tendency to increased obviously. Before the tensile test, the calculated values of retained austenite content in the CQ1-ART, CQ2-ART, and CQ3-ART samples are 49.2%, 62.0%, and 64.8%, respectively. It can be seen from Figure 2 and Figure 3b that the retained austenite content in the CQ3-ART sample reached the highest value (64.8%), and the grain size decreased from 0.62 μm for the CQ1-ART sample to 0.42 μm for the CQ3-ART sample, thus, it is considered that the stability of retained austenite increases with the decrease of grain size. However, combining the mechanical properties and XRD patterns of each experimental steel in Figure 3, it was found that the more retained austenite content with a stronger stability is not necessarily conducive to the improvement of overall performance.

### 3.2. Strengthening Mechanism of Nb-Mo Microalloy

Nb is easily precipitated with C and N in steel in the form of carbonitrides, and it can also exist in the form of solid solution. The addition of microalloying element Nb has a strong effect on grain refinement, phase transformation behavior, carbon enrichment in austenite and ferrite nucleation [28,29]. Generally, in order to optimize the role of Nb in steel, Mo is usually added to Nb-containing steel. Fu et al. [30] found that the solute atoms drag the effect of Nb in low-carbon Nb microalloy steel and the pinning effect of the NbC precipitates together hindering the growth of grains and the migration of grain boundaries. The pinning effect of NbC precipitates at high temperature plays a major role, while the solute drag effect of Nb does not, obviously. On the contrary, at relatively low temperature, Nb drag effect has a significant inhibitory effect on grain growth. Yuan et al. [31] found that the microalloying element Mo has a strong interaction with Nb, Mo may dissolve into the precipitated particles of Nb(C,N) when Nb(C,N) precipitated. Wang et al. [32] found that with increasing tempering temperature, Mo segregated in the outer layer of the carbide as well as Mo element reduced the diffusion rate of C and other alloy elements in matrix, jointly inhibiting the diffusion of Nb and V from matrix into the carbide and hindering the growth of carbide particles. Zhang et al. [33] believed that the entry of Mo atoms slightly reduced the lattice constant of NbC, while the addition of Mo significantly increased the yield strength of Nb-containing steel, as well as the addition of Mo increased the number of NbC particles smaller than 10 nm in size; moreover, (Nb,Mo)C has high thermal stability, and then exhibited high precipitation strengthening.

**Figure 4:**
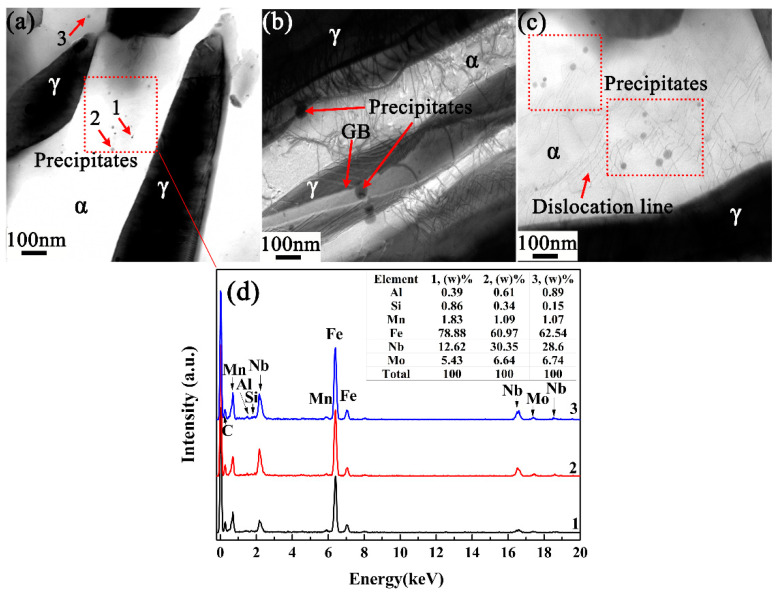
TEM picture of precipitates in CQ1-ART experimental steel: Nb/Mo precipitates on (**a**) ferrite (**b**) grain boundary, (**c**) dislocation line, respectively, and (**d**) precipitate TEM-EDS results.

Figure 4 shows the TEM micrograph of the Nb/Mo precipitates in the CQ1-ART sample. Figure 4a–c shows the Nb/Mo precipitates on the ferrite, grain boundary, and dislocation line of the CQ1-ART sample, respectively. There are a large number of Nb/Mo precipitates with a size of about 10~20 nm in ferrite, which hinder the dislocation movement and provide precipitation strengthening (see Figure 4a,d). Nb/Mo precipitates with a size of about 30~45 nm at the interface between ferrite and retained austenite have a drag effect on the grain boundary, which reduced the growth and coarsening speed of austenite and ferrite (see Figure 4b). Nb/Mo precipitates with a size of about 15~35 nm are pinned on the dislocation line, which hinders the dislocation movement in the deformation process. Thus, more external forces need to be added to overcome the pinning effect of precipitates on dislocation, thus the dislocation bypasses or cuts the precipitated phase. Finally, the dislocations need to bypass or cut through the precipitates to increase the strength of the matrix. It is worth noting that the grain size of the Nb/Mo precipitates increased in ferrite, dislocation lines, and γ/α grain boundaries [34].

Grain refinement produces two results [35]: On the one hand, the number of grains per unit volume increased as the grain size becomes finer, resulting in an increase in the sliding system, and then the probability of slippage under the action of external forces increases, resulting in the reduction of yield strength; on the other hand, the grain boundary increased with the grain size becoming finer, thus resulting in more places hindering the dislocation movement, and improving the yield strength. Therefore, grain refinement produces two comprehensive effects, and the key lies in which aspect plays the main role. Taking CQ1-ART sample as an example, its yield strength *σ_y_* is expressed by Equation (1) [36]:

(1) $$\sigma_{y}=\sigma_{0}+\sigma_{s}+\sigma_{g}+\sigma_{p}+\sigma_{d}\sigma_{y}=\sigma_{0}+\sigma_{s}+\sigma_{g}+\sigma_{p}+\sigma_{d}$$

where *σ*_0_, *σ_s_*, *σ_g_*, *σ_p_*, and *σ_d_* are the dislocation resistance of pure Fe at room temperature (57 MPa), solid solution strengthening increment, grain boundary strengthening increment, second phase precipitation strengthening increment, and dislocations, strengthen the increment, respectively. Wherein, the solid solution strengthening increment *σ_s_* is calculated by Equation (2) [36]:

(2) $$\sigma_{s}=4570\left[C\right]+67.7\left[P\right]+84\left[\mathrm{Si}\right]+32\left[\mathrm{Mn}\right]+11\left[\mathrm{Mo}\right]$$

in the Equation (2), [*X*] represents the mass fraction of solid solution *X* in the steel, where [C] and [Mo] are the contents after removing the precipitated C, respectively. P and Si mainly exist in the form of solid solution, while Mn, in addition to forming alloy cementite, also exists in the form of solid solution. It is concluded that the CQ1-ART sample solid solution strengthening increment *σ_s_* is 119 MPa.

The increase of fine grain strengthening σ_g_ is expressed by the Hall-Petch formula [36]:

(3) $$\sigma_{g}=k_{y}d^{-1/2}\sigma_{g}$$

where *k_y_* is the constant of proportionality, which is 17.4 MPa·mm^1/2^ in low carbon steel; *d* is the effective grain size, which is the smallest grain size that can hinder the sliding movement of dislocations and produce the effect of dislocation plugging. According to the statistics of Digital Micrograph software, the effective grain size for the CQ1-ART sample is 3.4 μm. Substituting it into Equation (3), the fine grain strengthening increment *σ_g_* is obtained as 299 MPa.

The second phase precipitation strengthening *σ_p_* is generally expressed as the external force required for the dislocation to bypass or cut through the second phase particles and continue to slip, which can be expressed by Equation (4) [36]:

(4) $$\sigma_{p}=0.1115G\frac{f_{V}^{1/2}}{d}In\left(2.417d\right)$$

where *G* is the shear modulus of pure Fe at room temperature and its value of 80,650 MPa, *f_V_* is the volume fraction of the precipitated phase and its value is calculated by Equation (5):

(5) $$f_{V}=f_{m}\frac{\rho_{Fe}}{100\rho_{MC}}$$

where *f_m_*, *ρ_Fe_*, and *ρ_MC_* are the mass fraction of MC precipitated phase, the density of Fe (7.875 g/cm^3^), and the density of MC precipitated phase, respectively. According to the XRD analysis results, it can be seen that the chemical formula of the MC phase in the CQ1-ART sample is (Nb_0.873_Mo_0.127_)C, and its density is assumed to be 7.803 g/cm^3^ equivalent to NbC. The second phase strengthening effect usually only considers particles smaller than 60 nm, so the second phase precipitation strengthening *σ_p_* of the CQ1-ART sample is calculated as 129 MPa.

There are a large number of dislocations in ferrite in CQ1-ART sample, and *σ_d_* is calculated by Equation (6)

(6) $$\sigma_{d}=2\alpha Gb\rho^{1/2}$$

where *α*, *b*, and *ρ* are the constant values related to the crystal structure (0.5), Burgers vector mode (0.248 mm), and dislocation density, respectively. According to the XRD results, the dislocation density of the CQ1-ART sample is 3.24 × 10^7^ mm^−2^, and the dislocation strengthening increment is 114 MPa. Parameters used for the yield strength calculation are shown in Table 1. In summary, the calculated CQ1-ART sample yield strength *σ_y_* is 718 MPa, which is slightly different from the measured yield strength of 705 MPa (combined with Figure 3). It is worth noting that the contribution to the yield strength in descending order is: fine grain strengthening, precipitation strengthening, solid solution strengthening, and dislocation strengthening.

### 3.3. Precipitation Behavior of Nano-Sized Carbides

**Figure 5:**
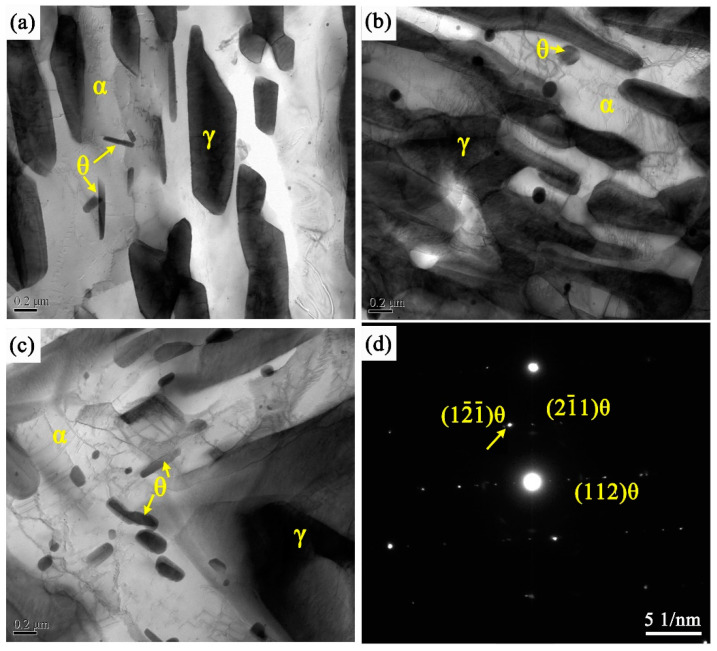
TEM picture of cementite in experimental steel (**a**) CQ1-ART, (**b**) CQ2-ART, (**c**) CQ3-ART, and (**d**) selected area electronic diffraction pattern (SAEDP).

Luo et al. [25,26] found that a large number of cementite particles will be formed in medium manganese steel during short-time intercritical annealing. As a non-thermodynamically stable phase, cementite is prone to dissolution during the annealing process in the intercritical region. Therefore, the author puts forward the hypothesis that these cementite particles are formed during heating. Under certain conditions, carbide particles can be used as new austenite grain nucleation points, which provides a new microstructure control idea for obtaining more austenite content to enhance the TRIP effect. It is possible to use carbide particles as the core of new austenite grains to increase the retained austenite content and stability by optimizing the amount, size, and composition of carbide precipitation before austenitization, rather than relying solely on the enrichment of carbon and manganese atoms. Generally, carbides become finer as the heating rate increases, while carbide particles with a fine size and uniform distribution are the prerequisites for their beneficial effects. Fu et al. [30] studied the strengthening effect of nano-scale cementite precipitates and found that there are a large number of cementite precipitates with a size less than 36 nm in Ti microalloyed high-strength weathering steel. The author found that the volume fraction of cementite less than 36 nm is 4.4 times that of TiC with the same size, and the precipitation of high volume fraction cementite has a stronger precipitation strengthening effect than nano-scale TiC. Figure 5 shows the TEM microstructure of cementite in three CQ-ART samples. Certain cementite particles were found in the three samples, most of which precipitated in the ferrite in rod-like or spherical morphology, with a diameter of 7–88 nm, a length of 110–260 nm, and a Mn content of 10.7–22.6 wt.%. Cementite particles are widely distributed in ferrite, mainly due to its nucleation at the moving martensite/austenite interface boundary. Spherical cementite particles are also formed near the austenite/ferrite interface in the austenite lath, with a size of about 80 nm. In short, manganese-rich cementite particles with a size of 10 nm can be formed after rapid heating to 690 °C and critical annealing for 1 h. It is considered that the precipitation of cementite particles with certain size and rich Mn will inevitably occur even if they are heated to the intercritical temperature rapidly.

**Figure 6:**
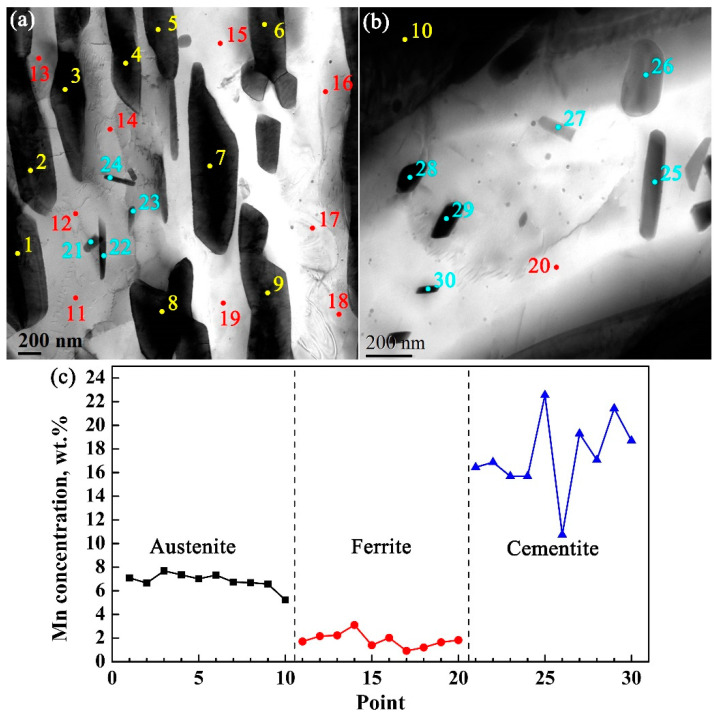
Mn content in different phases of CQ1-ART sample is determined by TEM-EDS, (**a**,**b**) TEM photos, (**c**) corresponding Mn concentration in different phases.

In order to analyze the distribution behavior of Mn in austenite, ferrite, and cementite more intuitively, the concentration of Mn in each phase is measured by TEM-EDS using CQ1-ART sample as an example, as shown in Figure 6. The concentration ranges of Mn measured in austenite, ferrite, and cementite are 5.23~7.69 wt.%, 0.92~3.11 wt.%, and 10.74~22.55 wt.%, respectively. It is considered that there is a significant Mn distribution behavior between three phases in the intercritical annealing process after rapid heating. The Mn content in rod-shaped or spherical cementite particles is significantly higher than that of ferrite and austenite (Figure 6c). Although cementite is thermodynamically unstable and easy to dissolve in the intercritical region, while the results show that cementite particles with a diameter of 7~88 nm formed after martensite was rapidly heated to the intercritical temperature for annealing, accompanied by a large amount of Mn distribution between cementite, ferrite, and austenite phases. It is worth noting that Al/Si elements in the experimental steel are insoluble in cementite, which hinders the formation and coarsening of cementite particles. In the intercritical annealing process, with the partitioning behavior of C and Mn, the C content in martensite decreases to a certain extent, which is called ferrite. However, the rapid distribution of C from martensite to cementite is owing to fact that part of the cementite inherits the initial Mn content of martensite, resulting in this rapid growth of cementite. Thus, the growth kinetics of cementite depends on the composition and size of its adjacent martensite phase, rather than the composition and size of the cementite core [26].

**Figure 7:**
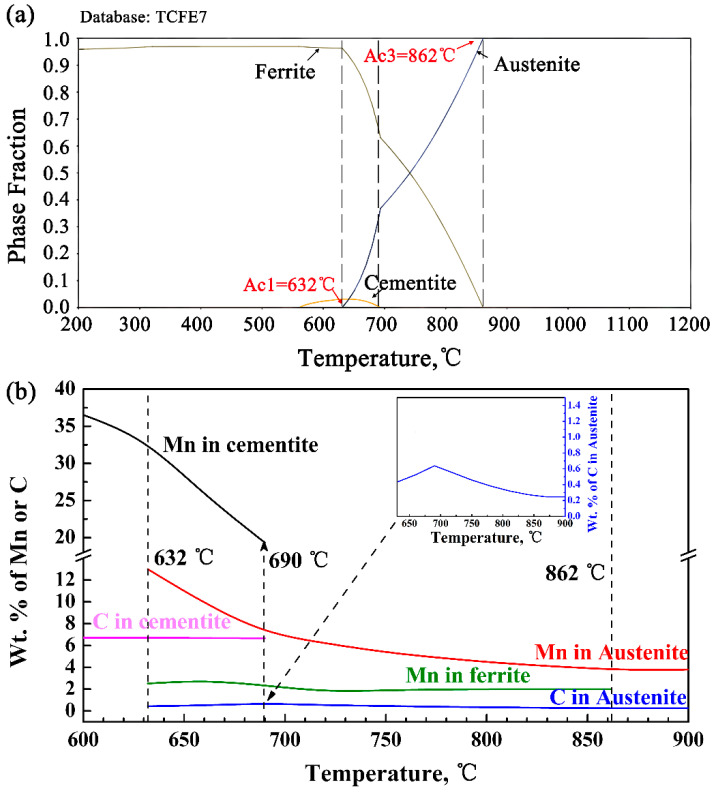
Equilibrium calculation of Thermo-Calc and TCFE7 database, (**a**) equilibrium phase diagram; (**b**) equilibrium content of elements in different phases.

Figure 7 shows the phase diagram calculated using thermo-Calc and TCFE7 databases and the element content under different stable equilibrium states. The intercritical region of the experimental steel is between f 632 °C and 862 °C, in which there are two phases of ferrite and austenite (see Figure 7a). The cementite dissolution temperature is 690 °C (Figure 7b), which corresponds to the maximum C content in equilibrium state of austenite (enlarged view in the upper right corner of Figure 7b). Theoretically, the equilibrium structure of the experimental steel at 690 °C is composed of 38% austenite and 62% ferrite, which contains 7.43 wt.% and 2.33 wt.% Mn, respectively. Cementite may still exist at 690 °C when it is in intercritical state, and the Mn and C contents in cementite are the lowest, which is 19.34 wt.% and 6.67 wt.% respectively. Thus, a certain amount of fine cementite was indeed found in the actual characterization process. The above results indicates that during the intercritical annealing process at the critical point of 690 °C, carbides with smaller sizes and lower Mn content will dissolve, and the decomposed C and Mn contents are conducive to the nucleation of austenite. Christian et al. [37] considered that carbide particles affect austenitizing kinetics through nucleation and growth rate. A large number of carbide/ferrite interface boundaries can be used as potential nucleation points, although there are fewer effective nucleation points, but the nucleation rate of austenite still varies with an increase in the area of the ferrite-carbide interface. In addition, the austenite growth rate is determined by the average diffusion distance of solute atoms, which is proportional to the distance between carbide particles [38]. Intercritical annealing at 690 °C temperature resulted in the existence of certain fine metastable carbide particles in the matrix of the experimental steel, which not only increased the nucleation rate of austenite, but also reduced the average diffusion distance of C, that is, the growth rate of austenite increased, and then more retained austenite content obtained.

## 4. Conclusions

In this paper, the strengthening mechanism and carbide precipitation behavior of Nb-Mo microalloy medium Mn steel were studied. The main conclusions of the present work are as follows:

(1) The (Nb, Mo) precipitates at the dislocation lines and the austenite-ferrite grain boundary in the experimental steel play a role in hindering the movement of dislocations and delaying the transformation of austenite to ferrite, which not only improves the strength but also refines the grains.
(2) The calculated yield strength value of the CQ1-ART sample is in good agreement with the measured value. Among them, the contribution to yield strength from large to small is: fine grain strengthening, precipitation strengthening, solid solution strengthening, and dislocation strengthening.
(3) Most of the cementite particles in the experimental steel precipitated in the ferrite in the form of rods or spheres, with a diameter of 7–88 nm, a length of 110–260 nm, and a Mn content of 10.7–22.6 wt.%. Carbide particles can be used as the nucleation sites of new austenite grains. At the same time, the C and Mn content decomposed from cementite with small size and low Mn content is conducive to austenite nucleation.

## Figures and Tables

**Table 1:** Parameters used for the yield strength calculation.

*σ*_0_	the dislocation resistance of pure Fe at room temperature (57 MPa)
*k_y_*	the constant of proportionality (17.4 MPa·mm^1/2^)
*G*	the shear modulus of pure Fe at room temperature (80,650 MPa)
*ρ_Fe_*	the density of Fe (7.875 g/cm^3^)
*α*	the crystal structure (0.5)
*b*	Burgers vector mode (0.248 mm)

## Data Availability

Not applicable.
